# A Large Comparative Cohort Study of Colonoscopy in the Elderly: Indications, Outcomes, and Technical Aspects

**DOI:** 10.7759/cureus.77619

**Published:** 2025-01-18

**Authors:** Fadi Abu Baker, Amir Farah, Amir Mari, Dorin Nicola, Rawi Hazzan, Oren Gal, Randa Taher

**Affiliations:** 1 Gastroenterology and Hepatology, Hillel Yaffe Medical Center, Hadera, ISR; 2 Gastroenterology and Hepatology, Ruth and Bruce Rappaport Faculty of Medicine, Technion - Israel Institute of Technology, Haifa, ISR; 3 Surgery, Medical College of Wisconsin, Milwaukee, USA; 4 Gastroenterology and Hepatology, EMMS Nazareth Hospital, Nazareth, ISR; 5 Gastroenterology and Hepatology, Liver Clinic, Clalit Health Services, Safed, ISR; 6 Gastroenterology and Hepatology, Azrieli Faculty of Medicine, Bar-Ilan University, Safed, ISR

**Keywords:** colonoscopy, elderly, endoscopy, gastroenterology, geriatrics, indications, outcomes, surgery

## Abstract

Introduction

Performing colonoscopy in the elderly is associated with unique challenges, including higher rates of comorbidities, limited physiological reserve, and procedural complexities. This study aimed to evaluate the technical aspects, indications, and outcomes of colonoscopy in the elderly, with an emphasis on indication-based diagnostic yield.

Methods

In this retrospective cohort study, we reviewed 35,000 consecutive colonoscopy procedures performed over a 12-year period on patients aged 50 years and older. Patients were categorized into three groups: very elderly (>80 years, n=3,434), elderly (65-80 years, n=13,783), and younger controls (50-64 years, n=17,959). Clinical and endoscopic findings were analyzed, with a focus on indication-specific outcomes.

Results

The most frequent indications for colonoscopy in the very elderly and elderly groups were anemia and rectal bleeding. Both elderly groups exhibited higher rates of inpatient procedures (49.2% and 20.9% vs. 9.6%; P<0.0001), inadequate bowel preparation (18.5% and 13.5% vs. 9.1%; P<0.0001), and anesthesiologist involvement in sedation (6.0% and 3.9% vs. 2.1%; P=0.03) but required lower doses of propofol sedation (4.5% and 5.4% vs. 7.9%; P=0.026). Colorectal cancer (CRC), polyps, and diverticulosis detection increased linearly with age. Colonoscopies performed for anemia or rectal bleeding yielded higher CRC and polyp detection rates, whereas constipation was associated with the lowest diagnostic yield.

Conclusion

This study highlights the technical challenges associated with performing colonoscopy in elderly patients, identifies indications with the highest diagnostic yield, and underscores the necessity of tailored bowel preparation protocols and an indication-driven approach to optimize the clinical utility of colonoscopy in this population.

## Introduction

Colonoscopy is the gold standard test for the investigation of lower gastrointestinal symptoms and is considered the modality of choice for colorectal cancer screening and surveillance [1,2]. Moreover, colonoscopy is an important therapeutic procedure in appropriate circumstances [3]. Because the incidence of colorectal pathology and symptoms increases with age, a large proportion of diagnostic, screening, and surveillance colonoscopies are performed on the elderly and very elderly population, defined as those above 65 and 80 years, respectively [4,5]. Indeed, several studies have demonstrated a significant increase in the risk of colorectal carcinoma (CRC) development in elderly patients compared to younger counterparts [6,7]. Moreover, increasing age is a risk factor for the development of precancerous lesions, as both adenomatous and advanced adenomatous have an increased prevalence and incidence in the elderly [5]. Thus, elderly patients may have higher overall diagnostic yields of colonoscopy when compared to younger controls.

The performance of screening colonoscopy in the elderly has attracted abundant research, and recommendations on screening in this group based on expected yield, although debated, have been issued in various relevant guidelines [8-10]. However, a great deal of colonoscopy procedures are performed for non-screening indications. Nevertheless, indication-based analysis of colonoscopy yield in this age group has attracted little research and focused on limited indications, particularly constipation and anemia [11-13].

Performing colonoscopy in elderly patients poses a unique set of challenges. In light of lower life expectancy and the frequent presence of comorbidities, the risks and benefits of colonoscopy should be carefully assessed to ensure that the potential benefits outweigh the risks and morbidity [14]. Besides, the procedural yield in this age group may be hampered by a higher frequency of poor bowel preparation and incomplete examinations [11]. Moreover, procedural setting and sedation process are major concerns in elderly patients that may have a direct impact on procedural performance, timing, and cost. We hypothesized that a higher percentage of procedures in the elderly are being performed in inpatient settings, and only a few studies have addressed the safety and efficacy of outpatient colonoscopy in very elderly patients, suggesting a higher cumulative incidence of post-colonoscopy complications in these patients [15]. Compared with younger patients, elderly patients were reportedly more susceptible to the adverse effects of all sedative drugs due to comorbidities and limited physiological reserve [16]. Despite the fact that several studies have shown that propofol sedation can be used safely in elderly patients, concerns still persist about the safety of sedation generally and propofol use specifically in the elderly [17,18]. Hence, strict monitoring is warranted, and administration of propofol by an anesthesia provider, rather than a gastroenterologist, may be preferred in many centers. Unfortunately, only a few reports have documented procedural settings and anesthesia-related data in this population, and therefore definite conclusions or recommendations are largely unavailable [19].

The current study investigates various aspects of colonoscopy procedures in elderly patients and provides an indication-based assessment of colonoscopy yield and outcome.

## Materials and methods

We performed a large cohort, retrospective study including consecutive patients who underwent colonoscopy procedures over a 12-year period (2008-2020) at the gastroenterology department of the Hillel Yaffe Medical Center, a university hospital in Israel. Exclusion criteria were mainly based on age and data availability. Patients were excluded if they were less than 18 years old at the time of the procedure or when a relevant data set was missing. We reviewed patients' electronic reports to extract relevant clinical data, including demographic details (age, sex, ethnicity), procedural setting (inpatient/outpatient), and procedure indications. Ethnicity was categorized based on the Israeli Central Bureau of Statistics (CBS) classification into religious ethnicity of the two main ethnic groups, Arabs and Jews. Moreover, relevant endoscopic data and information, including quality of bowel preparation (classified as adequate or inadequate based on the Aronchick Scale), depth of examination, endoscopic diagnosis, as well as sedation regimens, and providers, were collected. The patients were then divided into three different age groups: very elderly (above 80 years old), elderly (between 65 and 80 years old), and young patients (65≥ years and older) control groups. Procedural settings and indications, as well as sedation parameters and data, including dosage and rate of procedures performed by an anesthesia provider, were compared between groups. Overall outcomes, including the rate of complete examination, adequate bowel preparations, and endoscopic findings of CRC, polyps, diverticulosis, and inflammatory bowel disease, were also compared. Indication-based outcome analyses were evaluated, including per-indication CRC diagnosis rate and polyp detection rate (PDR).

Statistical analysis

Descriptive statistics in terms of mean, standard deviation, and percentage were used for all the parameters in the study. Differences within the elderly patients' group and between elderly age groups and the younger age control group were evaluated using Fisher's exact test for categorical parameters and the t-test for quantitative parameters. We performed multivariate analysis to identify predictors of increased PDR and CRC in the elderly groups. Multivariate logistic regression analysis was used to determine the effect of the independent parameters associated with CRC and PDR in terms of odds ratio and 95% confidence interval (95% CI). A value of P<0.05 was considered significant. All statistical analyses were conducted using the IBM SPSS Statistics for Windows, Version 25 (Released 2017; IBM Corp., Armonk, New York, United States).

## Results

**Figure 1:**
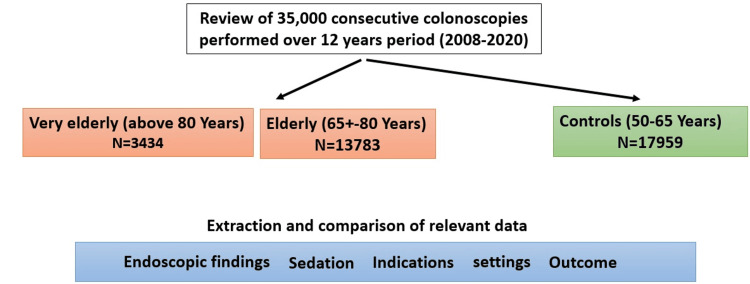
Study algorithm and major patient groups

Overall, we enrolled 17,217 elderly patients (13,783 in the elderly and 3,434 in the very elderly groups) compared to 17,959 younger controls aged 50-65 (Figure 1). No sex or ethnic differences were shown between the groups, reflecting our population's background distribution of gender and ethnicity.

The most prevalent indications for colonoscopy performance were anemia (27.9% and 16.8%), rectal bleeding (15.8% and 12.1%), and the investigation of abdominal pain (11.3% and 16.2%) for the very elderly and elderly groups, respectively (Table 1). Compared to controls, both elderly groups had significantly higher procedures performed in an inpatient setting (49.2% and 20.9% vs. 9.6%; P1,2<0.0001). Sedation dosage and provider differed between groups, as both elderly groups had higher anesthesiologists' involvement in procedural sedation (6% and 3.9% vs. 2.1%; P=0.03). Propofol as a monotherapy rather than in combination with other sedatives was increasingly used in elderly patients (31% and 17% vs. 7%; P1,2<0.05). Moreover, elderly patients were provided with lower doses of propofol and were in a lesser need for high-dose propofol sedation (4.5% and 5.4% vs. 7.9%; P=0.026).

**Table 1:** Baseline characteristics of study and control groups FOBT: fecal occult blood testing

Study Group	Controls (n=17,959)	65–80 years (n=13,783)	Above 80 years (n=3,434)	p-value
Sex (male)	9,132 (50.9%)	6,908 (50%)	1,677 (49%)	P1=0.19, P2=0.06
Ethnicity (Jew)	15,331 (85%)	11,807 (86%)	3,040 (87%)	P1=0.46, P2=0.168
Indication				
Abdominal pain	3,445 (19.2%)	2,226 (16.2%)	387 (11.3%)	P1,2<0.0001
Anemia	1,542 (8.6%)	2,313 (16.8%)	967 (27.9%)	P1,2<0.001
Positive FOBT	1,889 (10.5%)	1,362 (9.9%)	109 (3.2%)	P1,2<0.001
Rectal bleeding	2,952 (16.4%)	1,663 (12.1%)	543 (15.8%)	P1,2<0.001
Family history	2,144 (11.9%)	531 (3.9%)	12 (0.3%)	P1,2<0.0001
Screening	1,117 (6.2%)	373 (2.7%)	9 (0.3%)	P1,2<0.0001
Constipation	1,624 (9.0%)	1,605 (11.6%)	388 (11.3%)	P1,2<0.0001
Imaging finding	625 (3.5%)	709 (5.1%)	282 (8.2%)	P1,2<0.0001
Weight loss	455 (2.5%)	604 (4.4%)	261 (7.6%)	P1,2<0.0001
Sedation				
Anesthesiologist	395 (2.1%)	532 (3.9%)	210 (6.1%)	P1,2<0.05
Propofol monotherapy	1,288 (7%)	2,323 (17%)	1,064 (31%)	P1,2<0.05
Propofol average dose (mg)	160±14.25	132±11.7	110±8.92	P1,2<0.05
Propofol 200< (mg)	1,418 (7.9%)	745 (5.4%)	141 (4.1%)	P1,2<0.05
Inpatient setting (%)	1,723 (9.6%)	2,879 (20.9%)	1,619 (47.2%)	P1,2<0.0001

**Figure 2:**
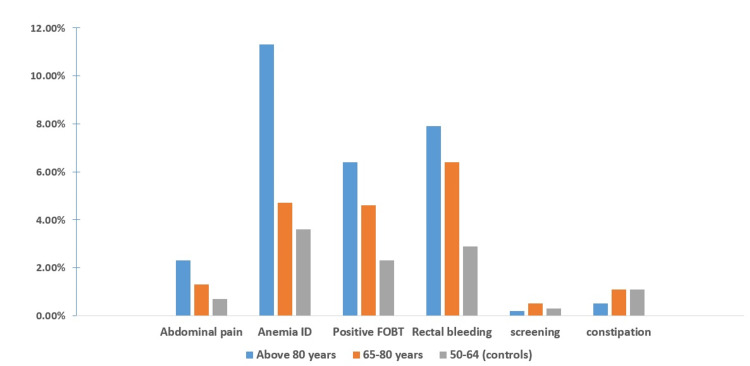
Colorectal cancer diagnosis per procedures' indications* *The differences between the colorectal cancer rate between both the elderly and control groups in iron-deficiency anemia, positive fecal occult blood testing (FOBT), and rectal bleeding indications were all significant (P<0.05), while the differences for the other indications were insignificant (P>0.05).

Endoscopic findings demonstrated significantly higher rates of inadequate bowel preparation (18.5% and 13.5% vs. 9.1%; P1,2<0.0001) and lower cecal intubation rates in very elderly and elderly groups, respectively, compared to controls (Table 2). Moreover, CRC (7.8% and 3.9% vs. 1.9%; P1,2<0.0001), polyps (34.1% and 29.4% vs. 21.4%; P1,2<0.0001) and diverticulosis (30.8% and 21.2% vs. 10.5%; P1,2<0.0001), while the inflammatory bowel disease (IBD) diagnosis rate didn't differ between groups, with almost 2% of patients over the age of 80 were diagnosed with IBD. On indication-based analysis of outcome, procedures performed for anemia investigation, rectal bleeding, and positive occult blood tests were associated with higher rates of CRC and polyp detection rates. In contrast, constipation and abdominal pain indications were associated with the lowest yield of colonoscopy investigation in the elderly groups (Figure 2). The multivariate analysis showed that besides the increasing age, indications of iron-deficiency anemia, rectal bleeding, and positive occult blood tests were predictors for increased polyps and CRC detection (Tables 3, 4). Male sex was a predictor of increased PDR (OR 1.516, 95% CI 1.44-1.59; P<0.001) but not increased CRC detection. Expectedly, poor bowel preparation was associated with reduced CRC and polyp detection rates.

**Table 2:** Major endoscopic findings in both study groups and controls IBD: inflammatory bowel disease

Age group	50-64 years	65-80 years	> 80 years	p-value
Inadequate preparation n(%)	1636 (9.1%)	1722 (13.1%)	629 (19.1%)	P^1,2^<0.001
Complete exams (%)	97.1%	94.2%	89.4%	P^1,2^<0.05
Polyp	3836 (21.4%)	4056 (29.4%)	1077 (34.1%)	P^1,2^<0.0001
Colorectal cancer	350 (1.9%)	535 (3.9%)	269 (7.8%)	P^1,2^<0.0001
IBD	472 (2.6%)	276 (2.2%)	76 (2.1%)	P^1,2^>0.05
Diverticulosis	1885 (10.5%)	2928 (21.2%)	1059 (30.8%)	P^1,2^<0.0001

**Table 3:** Predictors of polyp detection (multivariate analysis) FOBT: fecal occult blood testing

Variable	p-value	Odds ratio	95% confidence interval	
			Lower	Upper
Age (60-80 years)	< .001>	1.994	1.728	2.301
Age (above 80 years)	< .001>	3.583	3.004	4.275
Sex (Male)	< .001>	1.516	1.440	1.596
Iron-deficiency anemia	.050	1.179	1.000	1.391
Positive FOBT	.011	4.050	1.373	11.942
Rectal bleeding	.005	1.276	1.076	1.512
Constipation	< .001>	.340	.252	.458
Poor bowel preparation	< .001>	.638	.553	.734
Incomplete bowel examination	< .001>	.235	.203	.272

**Table 4:** Predictors of colorectal cancer detection (multivariate analysis) FOBT: fecal occult blood testing

Variable	p-value	Odds ratio	95% Confidence interval	
			Lower	Upper
Age (60-80 years)	< .001>	1.994	1.728	2.301
Age (above 80 years)	< .001>	3.583	3.004	4.275
Sex (Male)	.460	1.048	.926	1.185
Abdominal pain	< .001>	.259	.195	.345
Iron deficiency anemia	.005	1.189	1.09	1.391
Positive FOBT	.039	3.137	1.060	9.281
Rectal bleeding	.005	1.276	1.076	1.512
Constipation	< .001>	.340	.252	.458
Other indications	.769	.967	.774	1.208
Poor bowel preparation	< .001>	.638	.553	.734

## Discussion

Almost one-third of the colonoscopy procedures performed in our practice during the study period were performed on elderly patients. The majority of these procedures were performed to investigate overt symptoms (abdominal pain, rectal bleeding, constipation, and weight loss) or other specific clinical conditions, mainly iron deficiency anemia (IDA). Moreover, we showed that almost half of the very elderly and one-fifth of the elderly patients' procedures were performed in the inpatient setting. These figures reflect the considerable burden of endoscopic evaluation in this age group, regardless of screening indication. Thus, from an endoscopic point of view, we aimed to investigate various aspects of colonoscopy performance and outcome in this age group.

First, we provided comprehensive details on sedation regimens in elderly patients in real-life daily practice. Indeed, there is little evidence regarding sedation in the elderly, particularly very elderly patients, and this is an area where further research is required [20]. Few studies have shown that propofol sedation can be used safely in elderly patients [18,21]. The practice of gastroenterologist-administered propofol has also been shown to be safe in this age group [19]. As a center with more than ten years of experience with gastroenterologist-administered propofol sedation, data from the current study showed that we tend to give lower doses of sedation to elderly patients and use propofol as a monotherapy rather than a combination with other sedatives. Despite the fact that higher involvement of anesthesiologists in the sedation process was documented in elderly patients, the vast majority of elderly and very elderly patients in our study were sedated with gastroenterologist-administered propofol. Unfortunately, we couldn't obtain data on safety measures during and after the sedation process, and data on adverse events were unavailable and thus were not included.

Second, we demonstrated that elderly patients have a higher probability of inadequate bowel preparation, precluding complete procedures. This was translated into a decreased cecal intubation rate and was associated with lower polyp and CRC detection rates. Indeed, our findings reinforce existing literature, which lists older age among the risk factors for poor bowel preparation adequacy [22,23]. Worse mental and functional status may largely explain this. Not only does inadequate bowel preparation increase the miss rate of precancerous lesions, but it may also necessitate repeat procedures, a burden both to the patients and the health system. Thus, these patients should be targeted for further support, education, and other efforts to increase adequate preparation, such as stricter or tailored preparation regimens for the elderly, which warrant further evaluation.

Third, we showed a linear increase in colorectal CRC, polyps, and diverticulosis detection rates with age. These findings are straightforward and reinforce existing reports showing a substantial increase in the prevalence of detected adenomas and CRC with age [24,25].

Fourth, the indication-based analysis of the outcome showed interesting findings. We demonstrated that procedures performed for anemia investigation, rectal bleeding, or positive fecal occult blood tests were associated with higher CRC and polyp detection rates. In contrast, those performed for abdominal pain or constipation investigations were associated with the lowest yield of colonoscopy investigation in the elderly groups in this regard. These findings are of paramount importance, as almost 20% of colonoscopy procedures in our practice were performed to investigate abdominal pain and constipation. Moreover, reviewing the available evidence from the literature reveals that the performance of colonoscopy for the investigation of abdominal pain and constipation is controversial, and we could find very few studies suggesting that colonoscopy is not a helpful investigation in patients presenting solely with abdominal pain, as the diagnostic yield is poor [26]. However, despite our findings indicating an overall low yield, we demonstrated that the diagnostic yield has increased with age and that history-appropriate screening should be considered in the clinical encounter. Likewise, the literature review does not reveal a consensus regarding the role of colonoscopy for constipation as a sole symptom [27,28]. In comparison to previous studies, our study was unique in focusing on elderly patients and the direct comparison with an average-risk screening group. We showed that constipation as a sole indication had a lower risk of significant colonoscopy findings than average-risk screening. Thus, in the absence of other indications for colonoscopy, these patients may be managed with average-risk colonoscopy or fecal occult blood test screening, but further studies to confirm these findings are warranted.

Besides including a large cohort of elderly and very elderly patients, the strengths of the current study included the evaluation of various aspects of colonoscopy performance in real-life settings and the inclusion of a large control group.

Limitations of the current study are inherent in its retrospective and single-center nature. Moreover, integrating endoscopic and histopathology results was not always available, particularly for polyps. In addition, safety aspects of colonoscopy performance in the elderly were not part of the current study and weren't included.

## Conclusions

Our study highlights the importance of tailoring colonoscopy practices to the specific needs of elderly patients. By addressing procedural, sedation, and diagnostic challenges and focusing on high-yield indications, healthcare providers can maximize the benefits of colonoscopy while minimizing risks in this vulnerable population. Further efforts are required to refine patient selection, improve procedural outcomes, and ensure that colonoscopy remains a valuable tool in the diagnostic and therapeutic arsenal for elderly patients.
